# Supplementary material for: Ash1 and Tup1 dependent repression of the Saccharomyces cerevisiae HO promoter requires activator-dependent nucleosome eviction
Source: PLoS Genet. 2020 Dec 31;16(12):e1009133. doi: 10.1371/journal.pgen.1009133 (PMC7806131; doi:10.1371/journal.pgen.1009133)
Supplement: S1 Appendix — This appendix provides information on genes downstream of ATR peaks, that are potentially regulated by Ash1, Tup1, and Rpd3. (DOCX) [file pgen.1009133.s001.docx]

**S1 Appendix**

Identification of ORFs downstream of ATR peaks

Table S5 lists all genes potentially regulated by ATR peaks. Descriptions below highlight some of these genes that were not mentioned in the main text.

Pseudohyphal growth: Consistent with literature reporting Ash1 regulation of pseudohypthal growth, ATR peaks are present upstream of some genes involved in this process. These include ORFs encoding the pseudohyphal transcription factors Phd1 and Mit1 [1, 2], among others.

Meiosis and sporulation: Some proteins involved in meiosis have ATR peaks upstream of their ORFs, including the master meiotic regulator Ime1. Genes encoding spore wall assembly components and proteins needed for prospore formation are also potential ATR targets.

Previously identified Tup1-regulated genes: These include genes encoding the flocculins, a class of cell surface glycoproteins; mutants of tup1 display a flocculation phenotype consistent with misregulation of these genes [3]. ATR peaks are present upstream of *FLO9* and *FLO11*, as well as *BSC1*, which encodes a Flo11-like protein (Table S5). The *FLO10* gene may also be regulated by Ash1, Tup1 and Rpd3, as a peak is located within its long 5’ UTR (Table S5). Tup1 regulates *HXT* glucose transporter genes via Rgt1 [4], and a number of these ORFs (*HXT2*, *HXT6*, *HXT8*, *HXT9*, *HXT11*, *HXT13*, *HXT15*, and *HXT16*) also display localization of all three factors in the upstream region (in addition, the *HXT7* promoter region has an AT peak; Table S5). Other Tup1-regulated genes with upstream ATR peaks include *MFA2*, *ENA1*, *FET3* and *FTR1*. Ash1 may also be involved in recruiting Tup1 to *RNR2*, one of the Tup1-regulated ribonucleotide reductase genes involved in dNTP synthesis [5]. (Note that *RNR2* is not listed in Table S5, because the ATR peak is located over an uncharacterized ORF.) Tup1 regulates many genes that respond to a variety of cellular stresses, including hypoxia, DNA damage, and osmotic changes [6, 7]. A number of the possible ATR-regulated genes are responsive to these and/or other forms of stress (Table S5, ORFs in red type).

DNA binding transcription factors of several types: Of particular note are genes encoding other Tup1-recruiting DNA-binding transcription factors (*CIN5*, *NRG1*, *ROX1*, *PHD1*) as well as *ASH1* and *CYC8* (the partner protein for Tup1). The presence of ATR peaks within these promoters could indicate that pathways involved in Tup1-mediated repression can be auto-regulated.

Isomaltase genes: Four of the five isomaltase genes contain ATR peaks, while the fifth, *IMA1*, shows Tup1-only localization.

REFERENCES

1. Pan X, Heitman J. Sok2 regulates yeast pseudohyphal differentiation via a transcription factor cascade that regulates cell-cell adhesion. Mol Cell Biol. 2000;20(22):8364-72.

2. Cain CW, Lohse MB, Homann OR, Sil A, Johnson AD. A conserved transcriptional regulator governs fungal morphology in widely diverged species. Genetics. 2012;190(2):511-21.

3. Lipke PN, Hull-Pillsbury C. Flocculation of Saccharomyces cerevisiae tup1 mutants. J Bacteriol. 1984;159(2):797-9.

4. Ozcan S, Johnston M. Three different regulatory mechanisms enable yeast hexose transporter (HXT) genes to be induced by different levels of glucose. Mol Cell Biol. 1995;15(3):1564-72.

5. Klinkenberg LG, Webb T, Zitomer RS. Synergy among differentially regulated repressors of the ribonucleotide diphosphate reductase genes of Saccharomyces cerevisiae. Eukaryotic cell. 2006;5(7):1007-17.

6. Smith RL, Johnson AD. Turning genes off by Ssn6-Tup1: a conserved system of transcriptional repression in eukaryotes. Trends Biochem Sci. 2000;25(7):325-30.

7. Malave TM, Dent SY. Transcriptional repression by Tup1-Ssn6. Biochem Cell Biol. 2006;84(4):437-43.
