# Supplementary material for: Ash1 and Tup1 dependent repression of the Saccharomyces cerevisiae HO promoter requires activator-dependent nucleosome eviction
Source: PLoS Genet. 2020 Dec 31;16(12):e1009133. doi: 10.1371/journal.pgen.1009133 (PMC7806131; doi:10.1371/journal.pgen.1009133)
Supplement: S2 Appendix — This appendix describes experiments where potential Ash1 binding sites within the HO promoter were mutated and effects on HO expression were determined. (DOCX) [file pgen.1009133.s002.docx]

**S2 Appendix**

Attempts to identify Ash1 binding site locations within the *HO* promoter

An Ash1 DNA footprinting study suggested the sequence YTGAT as a possible motif for Ash1 binding [1]. We therefore examined all YTGAT motifs within the *HO* promoter and identified those for which at least four out of five positions of the YTGAT motif were conserved across multiple species of *Saccharomyces*, reasoning that conserved sites were more likely to be functionally relevant. Mutation of these motifs, either singly or in combinations, sometimes resulted in modest reduction of Ash1 and Tup1 binding at the *HO* promoter. However, none of them eliminated binding of Ash1 or reduced Tup1 binding to the level observed in an *ash1* mutant. Examination of the *HO* sequence near the Ash1 binding peaks also revealed identical 8-bp AATCGATG sequences at -1906 and -1223 that bear some resemblance to YTGAT. Mutation of this motif at -1223 reduced Ash1/Tup1 binding more than any other tested single motif substitution, but even in combination with additional mutations at other sites, binding of Ash1 was not eliminated, and Tup1 binding was not reduced to the level of an *ash1* mutant.

Motif 2, identified by MEME analysis (Fig S7) resembles the binding site for Mcm1 and was the most frequently identified motif identified within Ash1 peaks that is associated with another factor. *HO* was not one of the sequences that contained this motif. However, a directed search of the *HO* promoter for Motif 2 revealed sequences with some similarity to Motif 1/Mcm1, all outside the 100-bp window searched. One of these (located at -1244) lies just outside the 100-bp window for the Downstream Site summit and is thus still positioned relatively near the center of the predominant Ash1 peak (Fig S7, Fig 1A Downstream Site). Mutation of this -1244 motif reduced Ash1/Tup1 binding, but not as substantially as mutation of the -1223 site described above.

A number of Mcm1-interacting partners bind to sites adjacent to Mcm1 and compete with one another for association with Mcm1, participating in either activation or repression in different scenarios [2-4]. To investigate whether Ash1 could play a similar role as these proteins, we compared published ChIP-exo data for *in vivo* Mcm1 binding with the locations of Ash1 peaks [5]. Fifty-five Mcm1 sites are located within Ash1 peaks, representing 23% of the Ash1 peaks (Table S3). This suggests it is possible that Ash1 could contribute to Mcm1 regulation at some promoters, including such critical cell cycle genes as *CLB1-2*, *CLN1-3*, and *SWI4* (Table S5). However, not all Ash1 sites containing the Mcm1-like Motif 2 display Mcm1 binding, and some sites of Mcm1/Ash1 overlap do not have Motif 2 (Table S3). Therefore, the Ash1/Mcm1 relationship may be more complex than a simple model in which a portion of Ash1 sites are juxtaposed to Mcm1 sites. Other scenarios are possible, including a competition between Ash1 and Mcm1 for the same binding site, or a requirement of Mcm1 for Ash1 binding at some sites.

REFERENCES

1. Maxon ME, Herskowitz I. Ash1p is a site-specific DNA-binding protein that actively represses transcription. Proc Natl Acad Sci USA. 2001;98(4):1495-500.

2. Pramila T, Miles S, GuhaThakurta D, Jemiolo D, Breeden LL. Conserved homeodomain proteins interact with MADS box protein Mcm1 to restrict ECB-dependent transcription to the M/G1 phase of the cell cycle. Genes Dev. 2002;16(23):3034-45.

3. Kumar R, Reynolds DM, Shevchenko A, Goldstone SD, Dalton S. Forkhead transcription factors, Fkh1p and Fkh2p, collaborate with Mcm1p to control transcription required for M-phase. Curr Biol. 2000;10(15):896-906.

4. Darieva Z, Clancy A, Bulmer R, Williams E, Pic-Taylor A, Morgan BA, et al. A competitive transcription factor binding mechanism determines the timing of late cell cycle-dependent gene expression. Mol Cell. 2010;38(1):29-40.

5. Rossi MJ, Lai WKM, Pugh BF. Genome-wide determinants of sequence-specific DNA binding of general regulatory factors. Genome Res. 2018;28(4):497-508.
