# Supplementary figures and images for: Ash1 and Tup1 dependent repression of the Saccharomyces cerevisiae HO promoter requires activator-dependent nucleosome eviction
Source: PLoS Genet. 2020 Dec 31;16(12):e1009133. doi: 10.1371/journal.pgen.1009133 (PMC7806131; doi:10.1371/journal.pgen.1009133)

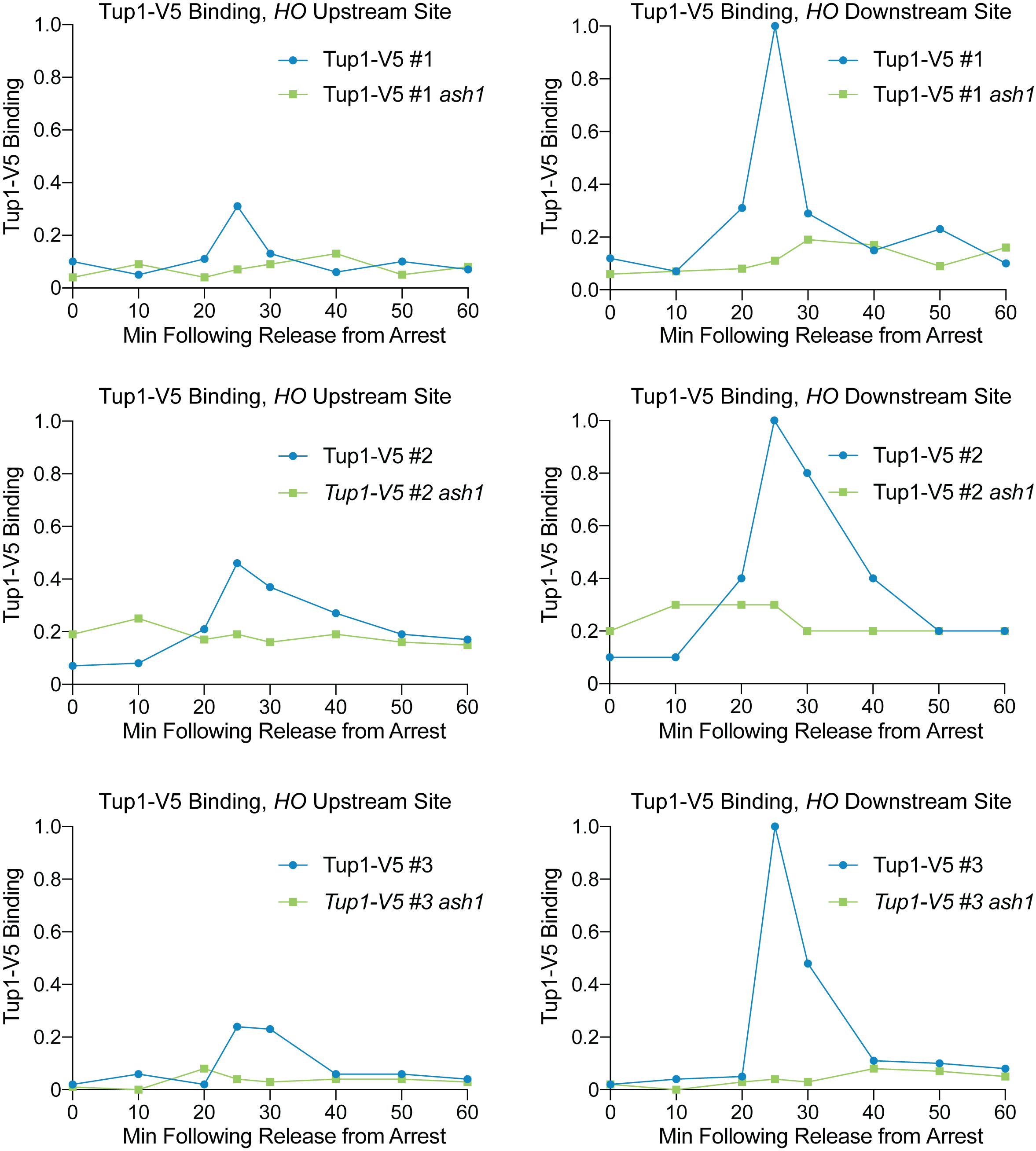

Supplement: S1 Fig — Data from Fig 1D is shown along with two additional replicates of the experiment. Binding of Tup1-V5 was measured by ChIP analysis with cells containing the GALp::CDC20 allele and synchronized by galactose withdrawal and readdition. The 0 min time point represents the G2/M arrest, before release with galactose addition. Cells were harvested at the indicated time points following release (x-axis), and samples were processed for ChIP analysis. Graphs show binding of Tup1-V5 in wild type (blue) and ash1 (green) cells, at the HO Upstream Site (left) and HO Downstream Site (right). Enrichment for each sample at HO was normalized to enrichment at an intergenic region on chromosome I (IGR-I) and to the corresponding input sample. (TIF) [file pgen.1009133.s003.tif]

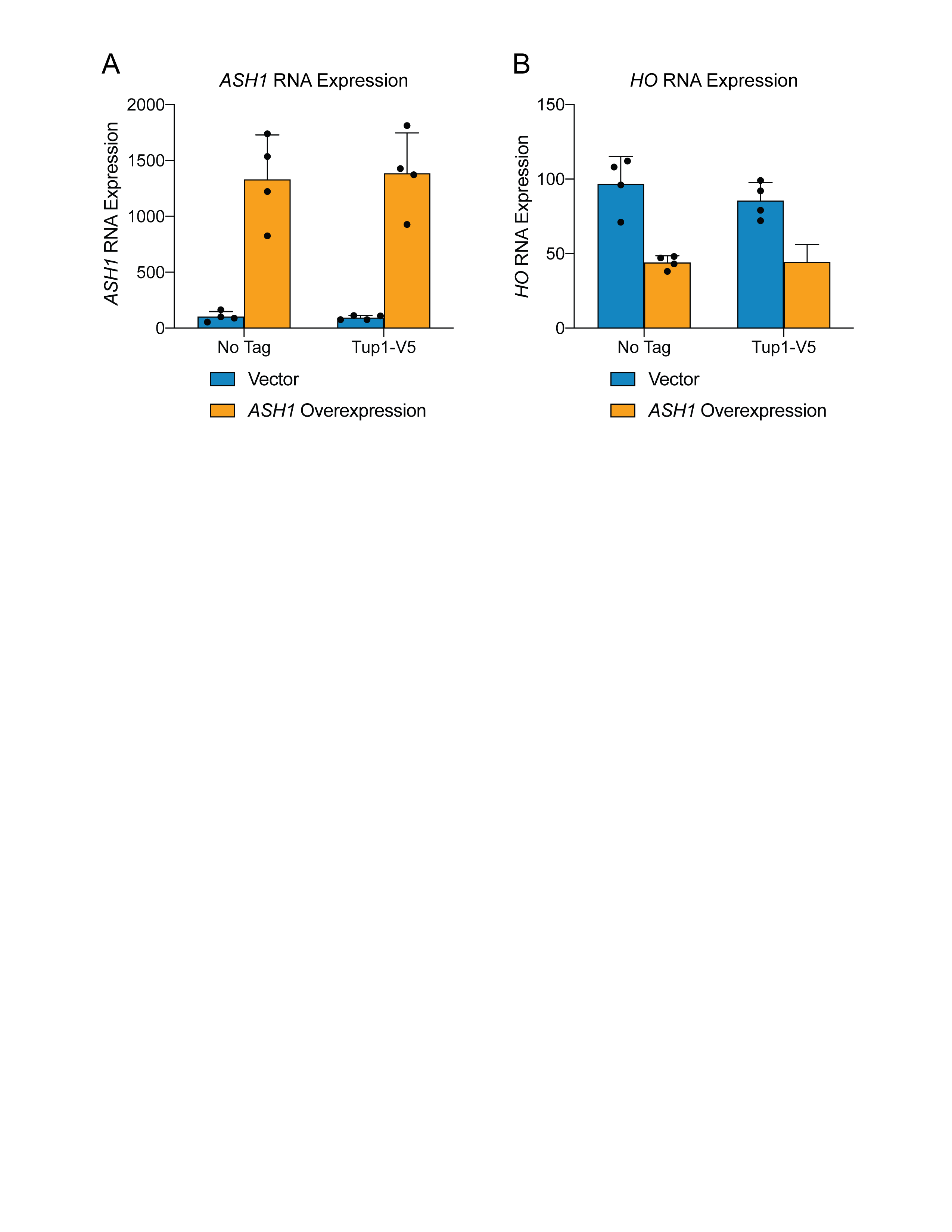

Supplement: S2 Fig — (A) A YEp-ASH1 multicopy plasmid results in increased ASH1 mRNA. ASH1 mRNA analysis under conditions of ASH1 overexpression, using cell samples identical to those in Fig 1E (Tup1-V5 ChIP analysis). Strains were transformed with a pRS426 YEp-URA3 vector, either empty (blue) or containing ASH1 (green). ASH1 mRNA levels were measured, normalized to RPR1, and expressed relative to wild type. Each dot represents a single data point, and error bars reflect the standard deviation. (B) A YEp-ASH1 multicopy plasmid results in decreased HO mRNA levels. HO mRNA analysis under conditions of ASH1 overexpression, using cell samples identical to those in Fig 1E (Tup1-V5 ChIP analysis). Strains were transformed with a pRS426 YEp-URA3 vector, either empty (blue) or containing ASH1 (green). HO mRNA levels were measured, normalized to RPR1, and expressed relative to wild type. Each dot represents a single data point, and error bars reflect the standard deviation. (TIF) [file pgen.1009133.s004.tif]

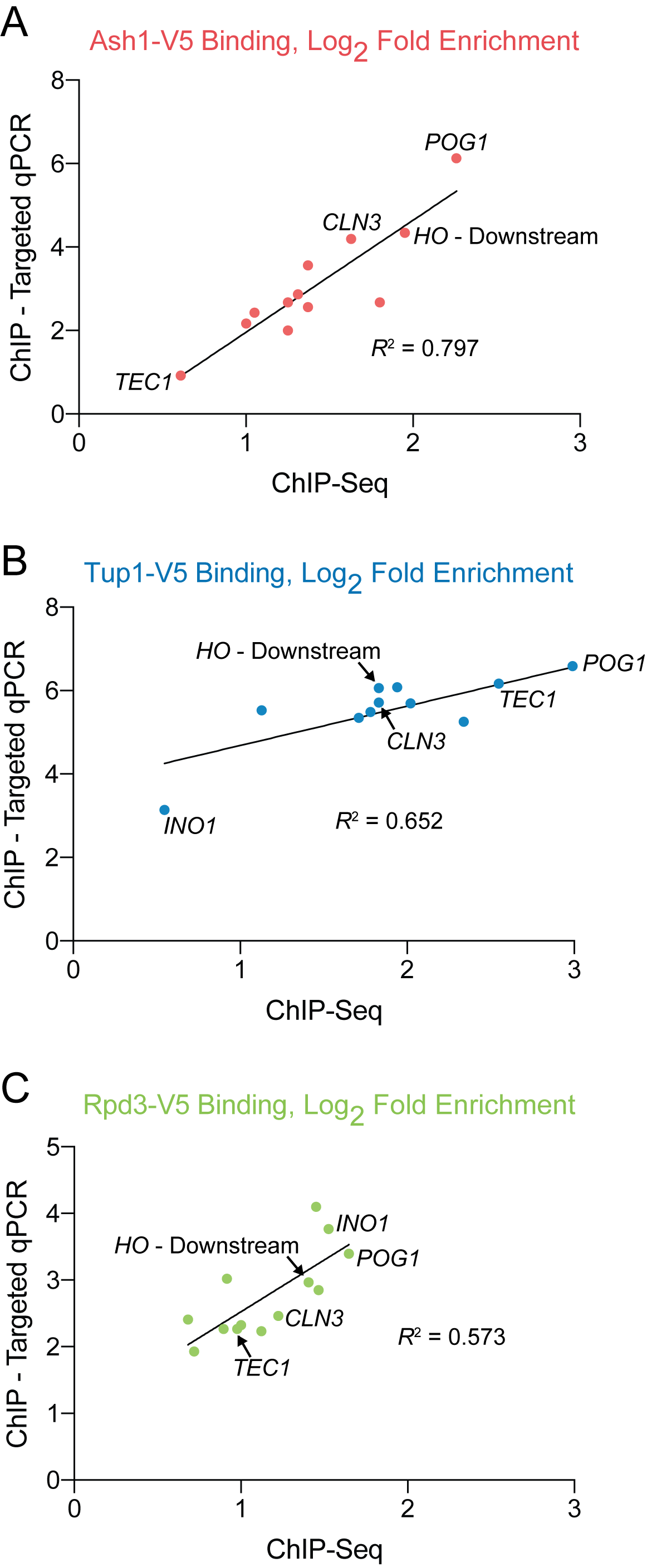

Supplement: S3 Fig — Correlation plots showing Ash1-V5 (A), Tup1-V5 (B) and Rpd3-V5 (C) log2 fold enrichment signals obtained via traditional ChIP (y-axis) and ChIP-Seq (x-axis). The genes tested are detailed in S4 Table. Gene common names identify some of the dots in the plots, including the HO Downstream site, CLN3 (used as positive control for Ash1-V5 ChIPs), TEC1 (used as positive control for Tup1-V5 ChIPs; very low Ash1-V5 binding), INO1 (used a positive control for Rpd3-V5 ChIPs; not bound by Ash1-V5), and POG1 (a high-scoring Ash1-V5 peak that shows co-localization with Tup1-V5 and Rpd3-V5). The R2 value obtained from linear regression analysis of each plot is shown. (TIF) [file pgen.1009133.s005.tif]

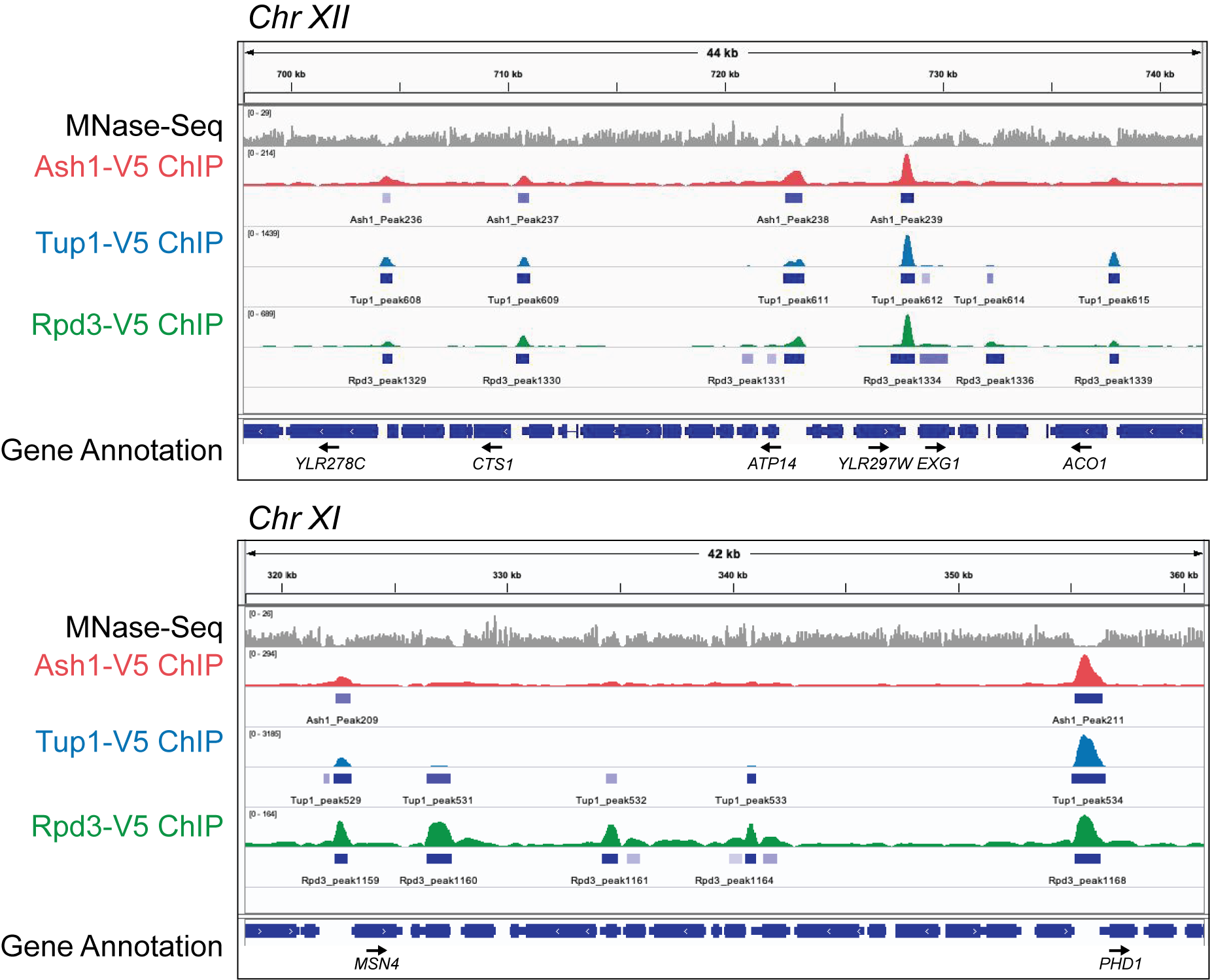

Supplement: S4 Fig — Additional snapshots of ChIP-Seq results from the Genome Browser IGV (Broad Institute), showing sequenced fragment pileups for the portion of the indicated chromosome, autoscaled for each factor independently (Refer to Fig 4B for another snapshot). The top track (gray) for each set shows MNase-Seq for nucleosome positioning reference. The colored tracks show ChIP-Seq results for Ash1-V5 (red), Tup1-V5 (blue) and Rpd3-V5 (green). The bottom track displays gene annotation. Gene names are indicated only for those with start sites downstream of a site of Ash1-V5, Tup1-V5, and Rpd3-V5 co-enrichment. (TIF) [file pgen.1009133.s006.tif]

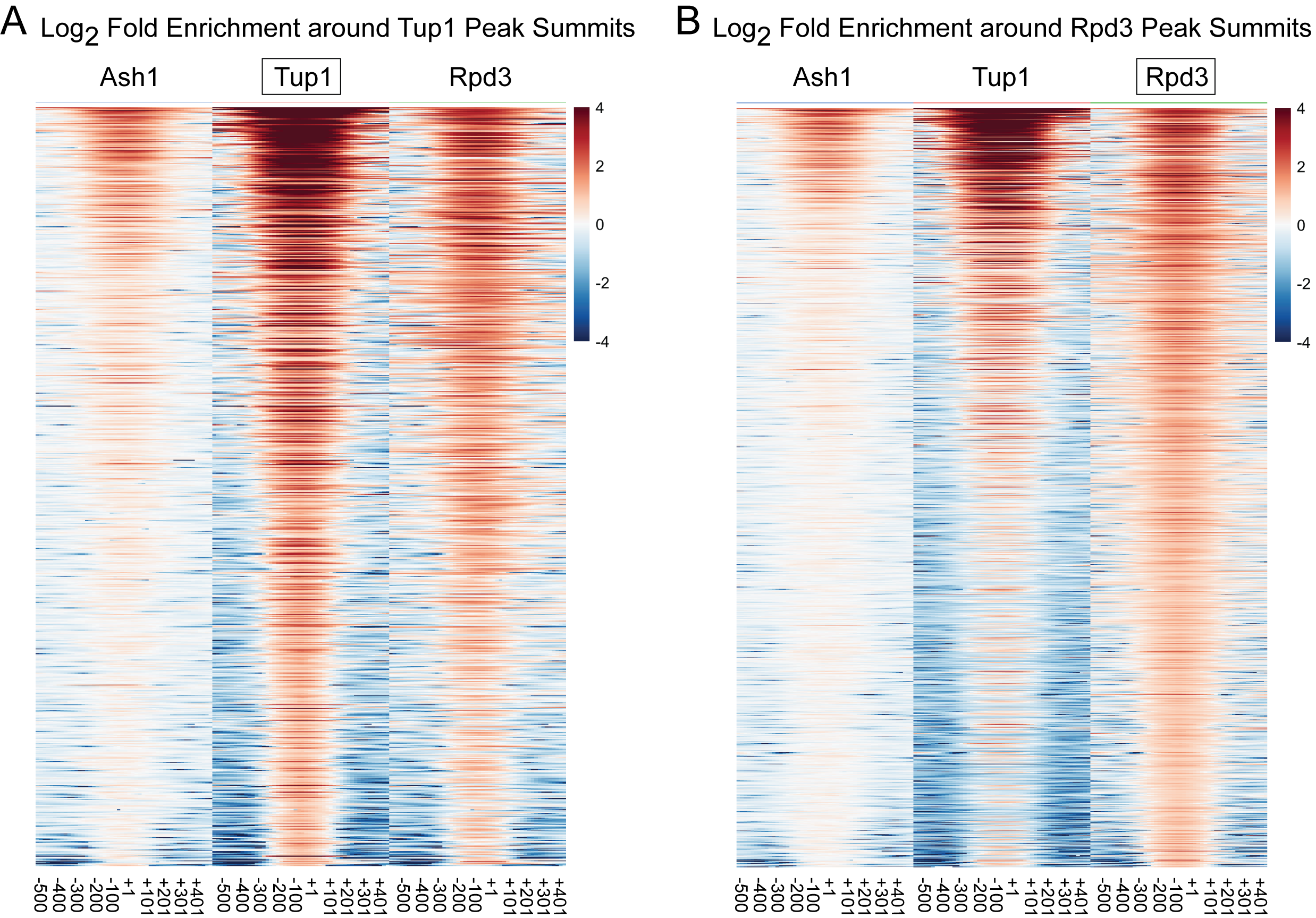

Supplement: S5 Fig — Heat maps depict the log2 fold enrichment of Ash1-V5, Tup1-V5 and Rpd3-V5 from -500 to +500 nucleotides relative to the center of each reference peak, in bins of 20-bp. The color scale at the right indicates the level of log2 fold enrichment for each factor. Each horizontal line depicts a single peak of enrichment. (A) Tup1 peaks (816) used as the reference. (B) Rpd3 peaks (1343) used as the reference. (TIF) [file pgen.1009133.s007.tif]

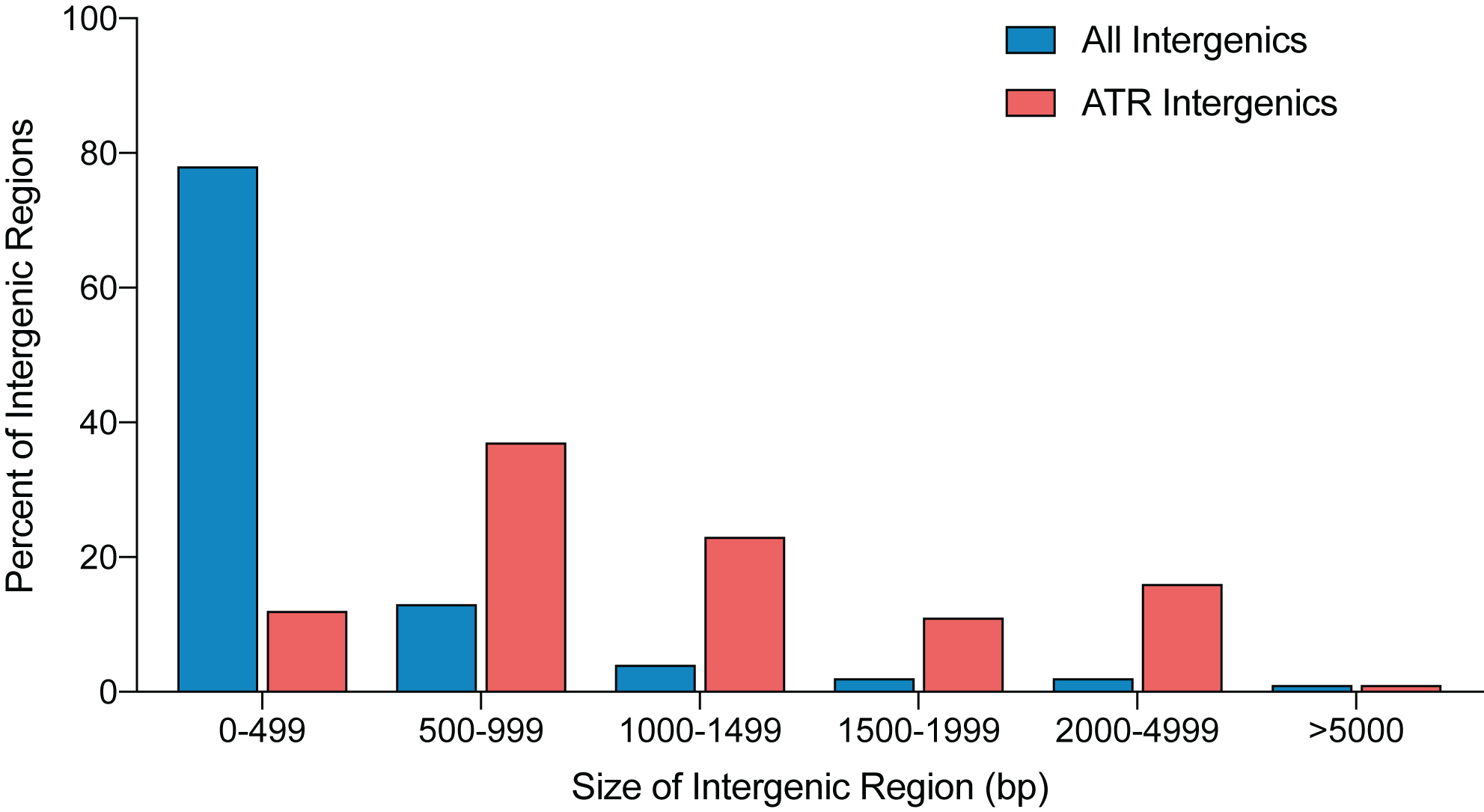

Supplement: S6 Fig — Shown is the percent of intergenic regions (y-axis) within each of six size categories of intergenic regions (x-axis). Distribution of genome-wide intergenic regions is shown in blue, and distribution of intergenic regions containing ATR co-localized peaks is shown in red. (TIF) [file pgen.1009133.s008.tif]

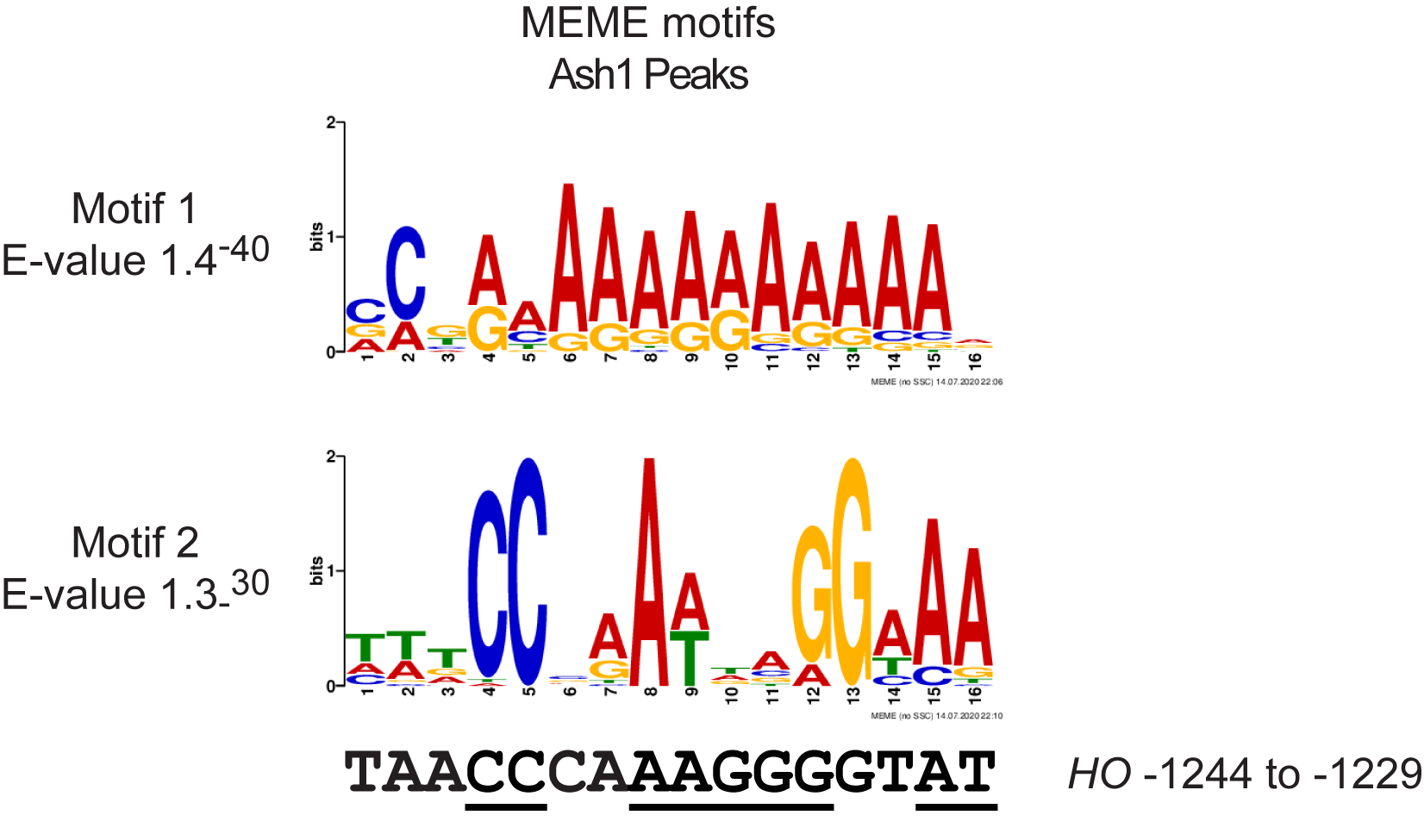

Supplement: S7 Fig — The top two motifs identified from MEME analysis of Ash1 peaks are shown. Motif 1 is found in 68 of the 250 Ash1 peaks, and Motif 2 was identified in 49 Ash1 peaks. Motif 2 resembles an Mcm1 motif [58,59]. The HO sequence from -1244 to -1229 is shown below Motif 2, to which it bears some similarity. Combined mutation of all positions in this region of the HO promoter (underlined) only modestly decreased Ash1 binding (S2 Appendix). (TIF) [file pgen.1009133.s009.tif]

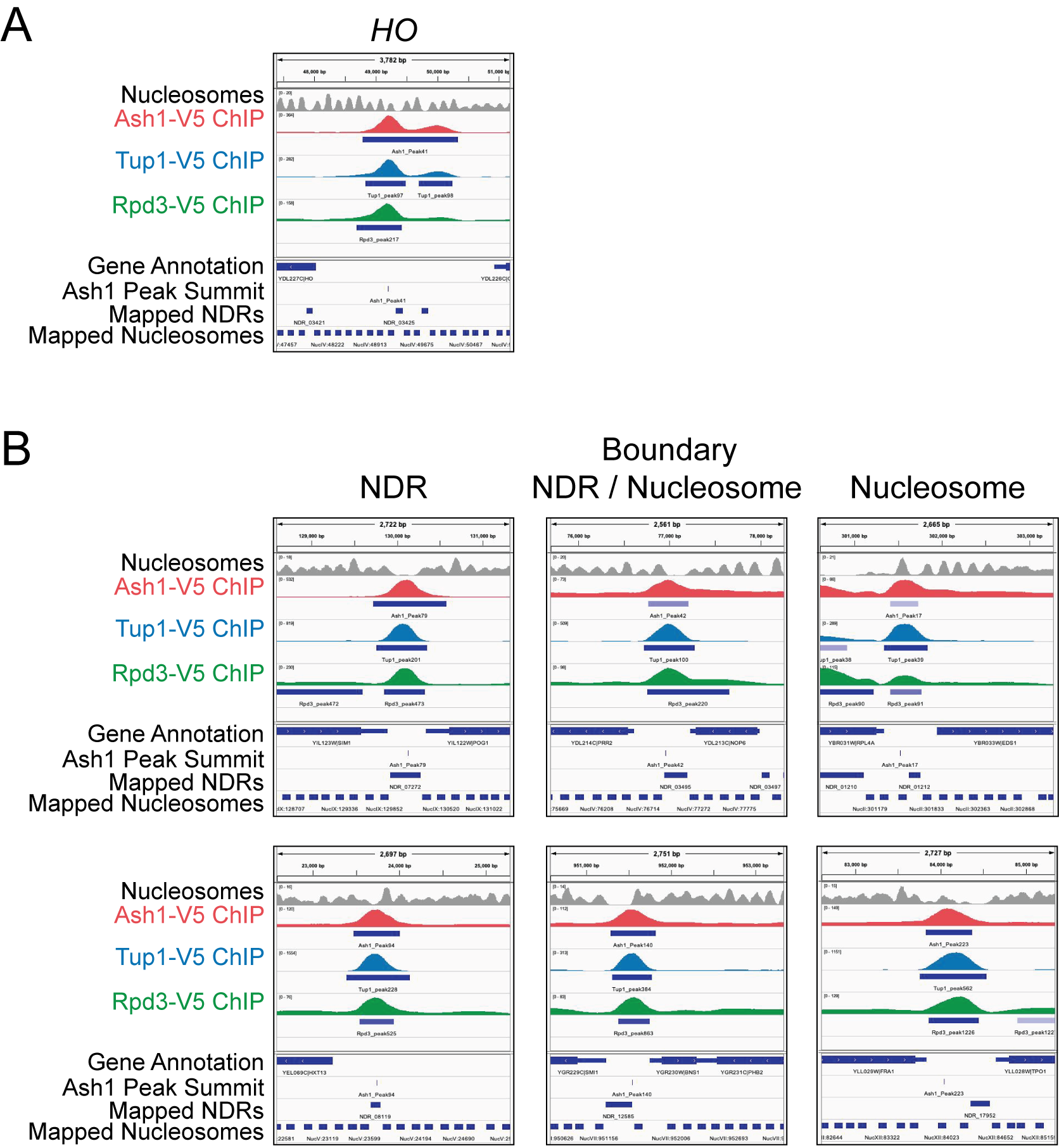

Supplement: S8 Fig — IGV genome browser snapshots of sequenced fragment pileups are shown to demonstrate the HO promoter (A) and two examples of ATR peaks from each category in Table 3 (B; NDR, NDR/Nucleosome Boundary and Nucleosome). Each factor was autoscaled independently. Tracks include: MNase-Seq nucleosome positions (gray), fragment density of Ash1-V5 (red), Tup1-V5 (blue) and Rpd3-V5 (green), annotations of peaks (beneath each fragment density track), gene annotation, position of the Ash1 peak summit, and mapped NDRs and nucleosomes (using the MNase-Seq data). (TIF) [file pgen.1009133.s010.tif]
