# Supplementary material for: Ash1 and Tup1 dependent repression of the Saccharomyces cerevisiae HO promoter requires activator-dependent nucleosome eviction
Source: PLoS Genet. 2020 Dec 31;16(12):e1009133. doi: 10.1371/journal.pgen.1009133 (PMC7806131; doi:10.1371/journal.pgen.1009133)
Supplement: S1 Table — (DOCX) [file pgen.1009133.s011.docx]

Supplemental Table S1. Strains used in this study.

| Figure 1A | | |
| --- | --- | --- |
| DY17937 | *MAT***a** *TUP1-V5::HIS3MX ade2 can1 his3 leu2 trp1 ura3* | |
| DY18407 | *MAT***a** *ASH1-V5::HIS3MX ade2 can1 his3 leu2 trp1 ura3* | |
| Figure 1B | | |
| DY150 | *MAT***a** *ade2 can1 his3 leu2 trp1 ura3* | |
| DY17937 | *MAT***a** *TUP1-V5::HIS3MX ade2 can1 his3 leu2 trp1 ura3* | |
| DY17935 | *MAT***a** *TUP1-V5::HIS3MX ash1::LEU2 ade2 can1 his3 leu2 trp1 ura3* | |
|  |  | |
| Figure 1C | | |
| DY17937 | *MAT***a** *TUP1-V5::HIS3MX ade2 can1 his3 leu2 trp1 ura3* | |
| DY17935 | *MAT***a** *TUP1-V5::HIS3MX ash1::LEU2 ade2 can1 his3 leu2 trp1 ura3* | |
| DY150 | *MAT***a** *ade2 can1 his3 leu2 trp1 ura3* | |
| Figure 1D |  | |
| DY18522 | *MAT***a** *ASH1-V5::HIS3MX GALp::CDC20::ADE2 ade2 can1 his3 leu2 trp1 ura3* | |
| DY17933 | *MAT***a** *TUP1-V5::HIS3MX GALp::CDC20::ADE2 ade2 can1 his3 leu2 trp1 ura3* | |
| DY17931 | *MAT***a** *TUP1-V5::HIS3MX ash1::LEU2 GALp::CDC20::ADE2 ade2 can1 his3 leu2 trp1 ura3* | |
| Figure 1E |  | |
| DY150 | *MAT***a** *ade2 can1 his3 leu2 trp1 ura3*  Transformed with pRS426, YEp-*URA3* vector (Christianson et al., 1992) | |
| DY150 | *MAT***a** *ade2 can1 his3 leu2 trp1 ura3*  Transformed with pAS174, *ASH1* in YEp-*URA3* (Sil and Herskowitz, 1996) | |
| DY17937 | *MAT***a** *TUP1-V5::HIS3MX ade2 can1 his3 leu2 trp1 ura3*  Transformed with pRS426, YEp-*URA3* vector (Christianson et al., 1992) | |
| DY17937 | *MAT***a** *TUP1-V5::HIS3MX ade2 can1 his3 leu2 trp1 ura3*  Transformed with pAS174, *ASH1* in YEp-*URA3* (Sil and Herskowitz, 1996) | |
| Figure 2B, C |  | |
| DY18852 | *MAT***a** *LYS2::lexA::HIS3 ade2 can1 his3 leu2 trp1 ura3* | |
| DY18848 | *MAT***a** *LYS2::lexA::HIS3 TUP1-V5::NATMX ade2 can1 his3 leu2 trp1 ura3* | |
| DY18892 | *MAT***a** *LYS2::lexA::HIS3 TUP1-V5::NATMX ASH1-LexA(DBD)-NLS-3xFLAG::HphMX ade2 can1 his3 leu2 trp1 ura3* | |
| Figure 3A | | |
| DY150 | *MAT***a** *ade2 can1 his3 leu2 trp1 ura3* | |
| DY1539 | *MAT***a** *rpd3::LEU2 ade2 can1 his3 leu2 trp1 ura3* | |
| DY17847 | *MAT***a** *tup1(H575Y) ade2 can1 his3 leu2 met15 trp1 ura3* | |
| DY18318 | *MAT***a** *rpd3::LEU2 tup1(H575Y) ade2 can1 his3 leu2 trp1 ura3* | |
| DY4394 | *MAT***a** *ash1::LEU2 ade2 can1 his3 leu2 trp1 ura3* | |
| Figure 3B | |  |
| yLB73 | *MAT***a** *ho-GFP-NLS-PEST::HIS3 MYO1::MCherry::HIS3MX can1 his3 leu2 trp1 ura3* |  |
| DY14797 | *MAT*α *ho-GFP-NLS-PEST::NatMX4 MYO1::MCherry::HIS3MX rpd3::LEU2 can1 his3 leu2 trp1 ura3* |  |
| DY18231 | *MAT***a** *ho-GFP-NLS-PEST::NatMX4 MYO1::MCherry::HIS3MX tup1(H575Y) can1 his3 leu2 trp1 ura3* |  |
| DY18724 | *MAT***a** *ho-GFP-NLS-PEST::NatMX4 MYO1::MCherry::HIS3MX rpd3::LEU2 tup1(H575Y) can1 his3 leu2 trp1 ura3* |  |
| DY15391 | *MAT***a** *ho-GFP-NLS-PEST::NatMX4 MYO1::MCherry::HIS3MX ash1::LEU2 can1 his3 leu2 trp1 ura3* |  |
| Figure 3C | |  |
| DY150 | *MAT***a** *ade2 can1 his3 leu2 trp1 ura3* |  |
| DY17937 | *MAT***a** *TUP1-V5::HIS3MX ade2 can1 his3 leu2 trp1 ura3* |  |
| DY19137 | *MAT***a** *TUP1-V5::HIS3MX sin3::LEU2 ade2 can1 his3 leu2 trp1 ura3* |  |
| Figure 4 | |  |
| DY18407 | *MAT***a** *ASH1-V5::HIS3MX ade2 can1 his3 leu2 trp1 ura3* |  |
| DY17937 | *MAT***a** *TUP1-V5::HIS3MX ade2 can1 his3 leu2 trp1 ura3* |  |
| DY17577 | *MAT***a** *RPD3-V5::HIS3MX ade2 can1 his3 leu2 trp1 ura3* |  |
| Figure 5B | |  |
| DY18407 | *MAT***a**  *ASH1-V5::HIS3MX ade2 can1 his3 leu2 trp1 ura3* |  |
| DY18924 | *MAT***a** *ASH1-V5::HphMX ho(del -1972 to -1826, replace CDC39)::KanMX(3') ade2 can1 his3 leu2 trp1 ura3* |  |
| DY18820 | *MAT***a** *ASH1-V5::HphMX ho(del -1288 to -1139, replace CDC39)::KanMX(3') ade2 can1 his3 leu2 trp1 ura3* |  |
| DY18930 | *MAT***a** *ASH1-V5::HphMX ho(del -1972 to -1826, replace CDC39 and del -1288 to -1139, replace CDC39)::KanMX(3') ade2 can1 his3 leu2 trp1 ura3* |  |
| Figure 5C | |  |
| DY17937 | *MAT***a** *TUP1-V5::HIS3MX ade2 can1 his3 leu2 trp1 ura3* |  |
| DY18908 | *MAT***a** *TUP1-V5::HIS3MX ho(del -1972 to -1826, replace CDC39)::KanMX(3') ade2 can1 his3 leu2 trp1 ura3* |  |
| DY18785 | *MAT***a** *TUP1-V5::HIS3MX ho(del -1288 to -1139, replace CDC39)::KanMX(3') ade2 can1 his3 leu2 trp1 ura3* |  |
| DY18914 | *MAT***a** *TUP1-V5::HIS3MX ho(del -1972 to -1826, replace CDC39 and del -1288 to -1139, replace CDC39)::KanMX(3') ade2 can1 his3 leu2 trp1 ura3* |  |
| DY17935 | *MAT***a** *TUP1-V5::HIS3MX ash1::LEU2 ade2 can1 his3 leu2 trp1 ura3* |  |
| Figure 5D | |  |
| DY150 | *MAT***a** *ade2 can1 his3 leu2 trp1 ura3* |  |
| DY18928 | *MAT***a** *ho(del -1972 to -1826, replace CDC39)::KanMX(3') ade2 can1 his3 leu2 trp1 ura3* |  |
| DY18824 | *MAT***a** *ho(del -1288 to -1139, replace CDC39)::KanMX(3') ade2 can1 his3 leu2 trp1 ura3* |  |
| DY18934 | *MAT***a** *ho(del -1972 to -1826, replace CDC39 and del -1288 to -1139, replace CDC39)::KanMX(3') ade2 can1 his3 leu2 trp1 ura3* |  |
| DY4394 | *MAT***a** *ash1::LEU2 ade2 can1 his3 leu2 trp1 ura3* |  |
|  |  |  |
| Figure 7A | |  |
| DY18522 | *MAT***a** *ASH1-V5::HIS3MX GALp::CDC20::ADE2 ade2 can1 his3 leu2 trp1 ura3* |  |
| DY18692 | *MAT***a** *SWI5-V5::HIS3MX GALp::CDC20::ADE2 ade2 can1 his3 leu2 trp1 ura3* |  |
|  |  |  |
| Figure 7B | |  |
| DY18407 | *MAT***a** *ASH1-V5::HIS3MX ade2 can1 his3 leu2 trp1 ura3* |  |
| DY18456 | *MAT***a** *ASH1-V5::HIS3MX ho(a3,b3)::KanMX(3') ade2 can1 his3 leu2 trp1 ura3* |  |
| DY17937 | *MAT***a** *TUP1-V5::HIS3MX ade2 can1 his3 leu2 trp1 ura3* |  |
| DY18227 | *MAT***a** *TUP1-V5::HIS3MX ho(a3,b3):KanMX(3') ade2 can1 his3 leu2 trp1 ura3* |  |
| DY13782 | *MAT***a** *UME6-Flag(3)::URA3::Flag(3) ade2 can1 his3 leu2 trp1 ura3* |  |
| DY18481 | *MAT***a** *UME6-Flag(3)::URA3::Flag(3) ho(a3,b3)::KanMX(3') ade2 can1 his3 leu2 trp1 ura3* |  |
| Figure 7D, E | |  |
| DY150 | *MAT***a** *ade2 can1 his3 leu2 trp1 ura3* |  |
| DY18407 | *MAT***a** *ASH1-V5::HIS3MX ade2 can1 his3 leu2 trp1 ura3* |  |
| DY18456 - | *MAT***a** *ASH1-V5::HIS3MX ho(a3,b3)::KanMX(3') ade2 can1 his3 leu2 trp1 ura3* |  |
| DY19045 - | *MAT***a** *ASH1-V5::HIS3MX ho::(a3,b3, Reb1 -1268 to -1262, Reb1 -1194 to -1189)::KanMX(3') ade2 can1 his3 leu2 trp1 ura3* |  |
|  |  |  |
| Figure S1 | |  |
| DY17931 | *MAT***a** *TUP1-V5::HIS3MX ash1::LEU2 GALp::CDC20::ADE2 ade2 can1 his3 leu2 trp1 ura3* |  |
| DY17933 | *MAT***a** *GALp::CDC20::ADE2 TUP1-V5::HIS3MX ade2 can1 his3 leu2 trp1 ura3* |  |
|  |  |  |
| Figure S2 | |  |
| DY150 | *MAT***a** *ade2 can1 his3 leu2 trp1 ura3*  Transformed with pRS426, YEp-*URA3* vector (Christianson et al., 1992) |  |
| DY150 | *MAT***a** *ade2 can1 his3 leu2 trp1 ura3*  Transformed with pAS174, *ASH1* in YEp-*URA3* (Sil and Herskowitz, 1996) |  |
| DY17937 | *MAT***a** *TUP1-V5::HIS3MX ade2 can1 his3 leu2 trp1 ura3*  Transformed with pRS426, YEp-*URA3* vector (Christianson et al., 1992) |  |
| DY17937 | *MAT***a** *TUP1-V5::HIS3MX ade2 can1 his3 leu2 trp1 ura3*  Transformed with pAS174, *ASH1* in YEp-*URA3* (Sil and Herskowitz, 1996) |  |

**References**

Christianson, T. W., R. S. Sikorski, M. Dante, J. H. Shero and P. Hieter (1992). Multifunctional yeast high-copy-number shuttle vectors. Gene **110**: 119-22.

Sil, A. and I. Herskowitz (1996). Identification of asymmetrically localized determinant, Ash1p, required for lineage-specific transcription of the yeast HO gene. Cell **84**: 711-22.
