# Supplementary material for: Ash1 and Tup1 dependent repression of the Saccharomyces cerevisiae HO promoter requires activator-dependent nucleosome eviction
Source: PLoS Genet. 2020 Dec 31;16(12):e1009133. doi: 10.1371/journal.pgen.1009133 (PMC7806131; doi:10.1371/journal.pgen.1009133)
Supplement: S2 Table — (DOCX) [file pgen.1009133.s012.docx]

Supplemental Table S2. Primers for ChIP and RT-qPCR Analysis.

| **ChIP Primers** | |  |
| --- | --- | --- |
| *HO* Promoter | |  |
| F3315 | *HO* -2318 (s) | CTGCAAACCAAAGAAAGATTGT |
| F3316 | *HO* -2318 (as) | TTTGAATTATGAGGCCCAGATAT |
| F3317 | *HO* -2228 (s) | TAATTCAAAAGACGGTGCCAT |
| F3318 | *HO* -2228 (as) | GTTGCAATGAGACCATTCTTTTC |
| F3319 | *HO* -2138 (s) | CATTGCAACACGTAAGGTTAAG |
| F3320 | *HO* -2138 (as) | CTCGTTCTCCCTACTATTTCACC |
| F3321 | *HO* -2043 (s) | GAGAACGAGTACCTGTAGTAAAAAGT |
| F3322 | *HO* -2043 (as) | ATGTTGGCGTTTGTCTCGAAC |
| F2083 | *HO* -1928 (s) | GAGTATTGTGTCATGTTCGAGACAAAC |
| F2084 | *HO* -1928 (as) | TTAAGTCCAAAGGCACAATTTTACG |
| F2085 | *HO* -1771 (s) | TTGATCTTTACCGTTTAGTTCCAAC |
| F2086 | *HO* -1771 (as) | GTAAAGCCTCCAGAACAGCTATG |
| F2087 | *HO* -1629 (s) | AAAGGCGGATCAAGATGTATGAAAG |
| F2088 | *HO* -1629 (as) | GGAACCATGTGATCTTACGTTGATATG |
| F2089 | *HO* -1528 (s) | TCCGAAAAGCAATTACTCTCTATGTT |
| F2090 | *HO* -1528 (as) | GCGATTGGGTATAATGAAGATTGTTA |
| F2091 | *HO* -1361 (s) | AAGCTAAGAATTTCACATGTTGTTG |
| F2092 | *HO* -1361 (as) | GTTGAGGTCTTTTCTATTTCTGATTG |
| F2093 | *HO* -1208 (s) | AATGCTGGAGCAAAAATTTCAATCAG |
| F3013 | *HO* -1208 (as) | TGGAGCCCCTCAGACATTAGC |
| F2115 | *HO* -1109 (s) | TCTACGGATGATCTGTGAGAA |
| F2116 | *HO* -1109 (as) | CTACGTTAAGACCTGTAACCGA |
| F2117 | *HO* -961 (s) | GAAAGAACCGCAGAGTGCTT |
| F2118 | *HO* -961 (as) | GAACCTGGTACGTATATTGTGGC |
| F2099 | *HO* -729 (s) | TCATACCCTGACTTGGCAAAC |
| F2100 | *HO* -729 (as) | CTTAAGCCCTGTGTAGGATTGATT |
| F2101 | *HO* -579 (s) | ATGCAGTTGAAGACATGTGCGTC |
| F2102 | *HO* -579 (as) | CATAGAAACAGGACTTGCGAACCC |
| F2103 | *HO* -455 (s) | ACGATTACCATGGAAATTAACGTACCT |
| F2104 | *HO* -455 (as) | TCTATGAAAATGAATTGTTGCTCTGC |
| F2105 | *HO* -349 (s) | GGTTTACGAAATGATCCACGAAAATC |
| F2106 | *HO* -349 (as) | TTTCACACCTAATAACGCCCAGC |
| F2119 | *HO* -191 (s) | ACCATTGGTACCTACTACTTTGAAT |
| F2120 | *HO* -191 (as) | GCCATTTAGAATAGGAATTGAATAC |
| F2083 | *HO* Upstream (s) | GAGTATTGTGTCATGTTCGAGACAAAC |
| F2084 | *HO* Upstream (as) | TTAAGTCCAAAGGCACAATTTTACG |
| F2093 | *HO* Downstream (s) | AATGCTGGAGCAAAAATTTCAATCAG |
| F3013 | *HO* Downstream (as) | TGGAGCCCCTCAGACATTAGC |
| F3661 | *HO*, Left (s) | GAATGGTCTCATTGCAACACG |
| F3662 | *HO*, Left (as) | ATGTTGGCGTTTGTCTCGAA |
| F1952 | *HO*, Right (s) | ATGTCTGAGGGGCTCCAACA |
| F1977 | *HO*, Right (as) | GATCGAACTTACTCAATAGC |
| F1093 | *HO* -1429 (s) | TATACCCAATCGCTGCGTGC |
| F1094 | *HO* -1158 (as) | AGCCGCCACGAATCAAACTT |
| F1916 | *HO*, Set 1 (s) | AGAGCTCATAATTCAAGCAA |
| F1936 | *HO*, Set 1 (as) | AACATAATTCCAGCACGCAG |
| F1998 | *HO*, Set 2 (s) | GCCGGTGCCTGCGATGAGAT |
| F2003 | *HO*, Set 2 (as) | TCGATTATTTGATACCCCTT |
| F2001 | *HO*, Set 3 (s) | CCTCAACAGTAATTAACCCA |
| F1094 | *HO*, Set 3 (as) | AGCCGCCACGAATCAAACTT |
| F1954 | *HO*, Set 4 (s) | AGTAATATTCCCAAGAAAGA |
| F1977 | *HO*, Set 4 (as) | GATCGAACTTACTCAATAGC |
| Positive Reference | |  |
| F2207 | *CLN3* (s) | TACATTCTGTGCTGGCGACC |
| F2212 | *CLN3* (as) | GCCAAGCGTTCAAACGAGAC |
| F1620 | *INO1* (s) | CGGAATCGAAAGTGTTGAAT |
| F1621 | *INO1* (as) | CGAAAGCTCCAATTTATATACGTC |
| F3490 | *TEC1* (s) | GCGTACGACTCAAAGAATTTCC |
| F3491 | *TEC1* (as) | TTGCTGATGGTAGCTACAGGTG |
| Negative Reference | |  |
| F1416 | IGR-I (s) | GTTTATAGCGGGCATTATGCGTAGATCAG |
| F1417 | IGR-I (as) | GTTCCTCTAGAATTTTTCCACTCGCACATTC |
| F1399 | IGR-V (s) | GGCTGTCAGAATATGGGGCCGTAGTA |
| F1400 | IGR-V (as) | CACCCCGAAGCTGCTTTCACAATAC |
| LexA Site-*HIS3* Reporter | |  |
| F3627 | LexA (s) | TGCCAGGAATTCACTAATCGC |
| F3628 | LexA (as) | GTTTTCCCAGTCACGACGTT |
| Genome |  |  |
| F2301 | *ELO1/CDC6* | AAATAGTGCAGCGCAGTG |
| F2315 | *ELO1/CDC6* | GAAACTAGTTGCTAGTCTGCA |
| F3750 | *LTE1* | ATTTCTTGAACCCTTTAGCCCAC |
| F3751 | *LTE1* | TGAGGATGGAGTCGGGTAGTAAT |
| F3754 | *MTR2/ASH1* | GCGCTTCCTCTTCTGTATTCCTT |
| F3755 | *MTR2/ASH1* | GGCTAGCGATGCGACAATAAAC |
| F3611 | *NRG2* | CACAATGGAGCCCAACAAAT |
| F3612 | *NRG2* | AGTCGGAAAATCGGAAAAGG |
| F3603 | *PCK1* | GAGAATATCGGGACGCTGAC |
| F3604 | *PCK1* | CCCGGAGCTTCAGTGATATG |
| F3605 | *PIL1* | TACGCAATTCGCATGTTACG |
| F3606 | *PIL1* | ATTGACGTGGCTGAACAACA |
| F3607 | *POG1* | TCAGGACATGAAGGCACAAC |
| F3608 | *POG1* | GATCCACAATTGCCCAAAAC |
| F3609 | *SCW10/FKS3* | CCCCAGAGATCTTCGGTCTA |
| F3610 | *SCW10/FKS3* | ACGTTTCAACGCGAATTTTT |
| F3758 | *TPO1* | TCAATCTTATAACCAGGCACCAGT |
| F3759 | *TPO1* | GAACCTATAATCCGTTGTGACCG |
| **RT-qPCR Primers** | |  |
| F1066 | *HO* (s) | AAATGGAGCGCTCTAAAGGAGAA |
| F1067 | *HO* (as) | CTAACCACAGACCAAGCATCCAA |
| F2430 | *RPR1* (s) | CACCTATGGGCGGGTTATCAG |
| F2431 | *RPR1* (as) | CCTAGGCCGAACTCCGTGA |
| F1693 | *ASH1* (s) | AAGTATTCGTTTCATTCGCTCAA |
| F1694 | *ASH1* (as) | CGGACGATAGCCTGTCTAACAA |
| Primers are arranged in pairs, with sense (s) and anti-sense (as) strands indicated. | | |
|  |  |  |
|  |  |  |
|  |  |  |
