# Supplementary material for: Ash1 and Tup1 dependent repression of the Saccharomyces cerevisiae HO promoter requires activator-dependent nucleosome eviction
Source: PLoS Genet. 2020 Dec 31;16(12):e1009133. doi: 10.1371/journal.pgen.1009133 (PMC7806131; doi:10.1371/journal.pgen.1009133)
Supplement: S4 Table — (DOCX) [file pgen.1009133.s014.docx]

Supplemental Table S4. Analysis of Ash1, Tup1, and Rpd3 Binding to a Subset of Diverse Promoters.

| Genes^a^ | Chr | Location^b^ | Ash1 | | Tup1 | | | | | Rpd3 | | |
| --- | --- | --- | --- | --- | --- | --- | --- | --- | --- | --- | --- | --- |
|  |  |  | ChIP-Seq log_2_FE | Occupancy^c^ | ChIP-Seq log_2_FE | Occupancy^c^ | WT / *ash1*^d^ | *ASH1* Overexp / Vector ^d^ | WT / *sin3* ^d^ | ChIP-Seq log_2_FE | Occupancy^c^ | WT / *ash1* ^d^ |
| *HO* Upstream | IV | NS | 1.95 | 7.9 ± 3.1 | 1.18 | 23 ± 2.7 | 3.4 | 4.6 | 0.8 | NA | 3.8 ± 1.5 | 1.7 |
| *HO* Downstream | IV | NUC | 1.95 | 20.3 ± 4.1 | 1.83 | 62 ± 13 | 5.9 | 7.4 | 0.9 | 1.41 | 7.8 ± 1.3 | 2.4 |
| *CLN3* | I | NDR | 1.63 | 18.3 ± 3.4 | 1.83 | 53 ± 3.8 | 2.9 | 3.4 | 0.8 | 1.22 | 5.5 ± 2.1 | 1.8 |
| *ELO1 / CDC6* | I | NDR | 1.31 | 7.3 ± 0.6 | 1.71 | 39 ± 6.5 | 3.3 | 2.7 | 0.7 | 0.92 | 8.1 ± 1.6 | 2.0 |
| *INO1* | X | NS | NA | 1.3 ± 0.7 | 0.547 | 8.8 ± 1.4 | 1.3 | 1.1 | 0.7 | 1.53 | 13.6 ± 2.6 | 1.2 |
| *LTE1* | I | NUC | 1.25 | 4.0 ± 0.7 | NA | 6.6 ± 3.2 | 1.6 | 1.0 | 1.7 | 1.45 | 17.1 ± 2.7 | 4.8 |
|  |  |  |  |  |  |  |  |  |  |  |  |  |
| *MTR2 / ASH1* | XI | NDR | 1.80 | 6.4 ± 0.8 | 1.94 | 67 ± 14 | 5.4 | NA | 0.9 | 1.00 | 5.0 ± 1.6 | 2.1 |
|  |  |  |  |  |  |  |  |  |  |  |  |  |
| *NRG2* | II | NUC | 1.37 | 5.9 ± 0.7 | 1.13 | 44 ± 6.0 | 1.9 | 2.2 | 1.4 | 0.72 | 3.8 ± 1.5 | 1.7 |
|  |  |  |  |  |  |  |  |  |  |  |  |  |
| *PCK1* | XI | NDR | 1.37 | 11.8 ± 1.9 | 2.02 | 50 ± 4.8 | 1.7 | 1.8 | 0.2 | 0.90 | 4.8 ± 1.7 | 1.8 |
| *PIL1* | VII | NDR | 1.25 | 6.4 ± 0.9 | 1.78 | 43 ± 6.8 | 2.1 | 3.4 | 0.5 | 0.68 | 5.3 ± 1.7 | 1.9 |
| *POG1* | IX | NDR | 2.26 | 70 ± 36 | 2.99 | 93 ± 10 | 2.4 | 2.1 | 0.9 | 1.65 | 10.5 ± 2.5 | 2.4 |
| *SCW10 / FKS3* | XIII | NDR | 1.05 | 5.4 ± 1.4 | 2.34 | 37 ± 4.9 | 1.5 | 1.9 | 0.5 | 1.47 | 7.2 ± 0.7 | 1.5 |
|  |  |  |  |  |  |  |  |  |  |  |  |  |
| *TPO1* | XIII | NUC | 1.00 | 4.5 ± 0.6 | 3.44 | 88 ± 21 | 1.5 | 2.3 | 1.4 | 1.12 | 4.7 ± 1.5 | 1.7 |
| *UBC4 / TEC1*^e^ | II | NDR | 0.61 | 1.9 ± 0.7 | 2.55 | 68 ± 11 | 1.4 | 2.0 | 3.7 | 0.98 | 4.8 ± 1.7 | 1.9 |

^a^ Only the genes that are downstream of binding peaks are indicated. If two genes are shown, the peak is between divergent promoters.

^b^ NS = Not Scored (Not an ATR Peak); NUC = peak over nucleosome; NDR = peak over NDR

^c^ Measured by traditional ChIP, relative to input sample and No Tag control. Average of 3 biological replicates ± standard deviation.

^d^ Measured by traditional ChIP. Ratios were calculated using the average of 3 biological replicates.

^e^ In the text and figures, this peak is simply listed as “*TEC1*” for simplicity.
