# Supplementary material for: Ash1 and Tup1 dependent repression of the Saccharomyces cerevisiae HO promoter requires activator-dependent nucleosome eviction
Source: PLoS Genet. 2020 Dec 31;16(12):e1009133. doi: 10.1371/journal.pgen.1009133 (PMC7806131; doi:10.1371/journal.pgen.1009133)
Supplement: Attachment — Submitted filename: Response to Reviewers_4.docx [file pgen.1009133.s017.docx]

**Response to Reviewer Comments**

**PGENETICS-D-20-01462**

Parnell, Parnell, Yan, Bai, and Stillman

Ash1 and Tup1 Dependent Repression of the Saccharomyces cerevisiae *HO* promoter Requires Activator-Dependent Nucleosome Eviction

Referee comments are in black. Author responses are in blue.

We thank the editor and the reviewers for their thoughtful comments on our paper. The reviewers’ comments have been extremely helpful and have led to a much improved manuscript.

Reviewer #1’s comments:

1) Results for an untagged control are presented for standard ChIP but apparently this control was not performed for ChIP-seq experiments. As I imagine the ChIP-seq experiments were performed in Dr. Bai’s lab, I wonder if she has results from any untagged control using the anti-V5 (or other) antibodies that could be shown together with the heat maps in Figure 6? I think it is not likely the results will change but it would be a nice control if available

We are rather confused by the reviewer’s comment. Perhaps the reviewer meant to refer to Figure 4C, and he/she erroneously assumed the ChIP-qPCR was done in one lab and the ChIP-Seq in a different lab. However, the ChIP-qPCR and the ChIP-Seq were performed in the same lab, using the same methodology. Untagged controls were included in preparation of samples for ChIP-Seq, and these were confirmed negative by qPCR prior to sending the Ash1-V5, Tup1-V5, Rpd3-V5 samples for ChIP-Seq. In addition, other evidence suggests these ChIP-Seq peaks are legitimate. First, the Tup1 ChIP data agrees with that of K. Stuhl’s lab (Wong and Struhl, 2011 Genes Dev. 25:2525); although these two ChIP-Seq data sets were not formally analyzed, it is clear that they are largely the same when examined via a genome browser. Second, based on the literature, we would have predicted that Tup1 and Rpd3 proteins show substantial overlap, which they do. Third, peaks at *HO* are at identical positions in the standard ChIP-qPCR and the ChIP-Seq (Fig 1A).

2) State in Figure 1 legend that replicates are shown in Fig S1.

The presence of replicates in Fig S1 is now indicated in the Fig 1 legend.

3) Table S5: It would be helpful to provide p-values for enrichment for the various categories shown. What is the source of information for stress responsive genes and for the other categories? I assume they correspond to standard Gene Ontology categories?

Table S5 had been revised to provide additional information about the categories and GO terms. The previous version was not compiled using GO terms, but used empirically determined categories. GO term analysis did not identify some of the categories that could be determined empirically, possibly because some categories have small numbers of genes and therefore would not pass a threshold p-value. The revised version has a few GO term categories shown (the most predominant and/or most general ones) but also contains some smaller empirically determined categories.

4) Line 598, “switch” should be “switches”

This typo has been fixed.

5) Ref 62 is incomplete-in press?

The reference to this In Press paper has been corrected. The paper has just been published.

6) Methods-presumably perfetto delitto was used for nucleosome replacements? This (or whatever method was used) should be stated.

The text has been re-written to clarify that the *delitto perfetto* method was used.

Reviewer #2’s comments:

1. The nucleosome positions on the HO promoter are derived from MNase-seq data from another study, presented in Fig. 1A. They are critical for the interpretation of the data, but the nucleosome positions were determined using log-phase cells and are therefore averaged over the cell cycle. Since Ash1 binding is cell cycle-dependent, it is possible that the nucleosome positions on the HO promoter change during the cell cycle and that their average positions are misleading. In addition, there is the complication that Ash1 may bind only in daughter cells and that the chromatin structures of mother and daughter cells also differ. The question that arises is whether the Ash1 binding site is always in a nucleosome, except when activated, as proposed? If Ash1 binds at HO for only ~10 minutes of the cell cycle and only in daughter cells, then a transient NDR where Ash1 binds might be expected in only perhaps 5% of cells in a log-phase population, which would be very difficult to detect with any certainty in MNase-seq data.

We agree with the author that MNase-seq data from a log-phase culture may not reflect what is happening in mother and daughter cells during the cell cycle. A sentence indicating this uncertainty has been added to the Results. In terms of the cell cycle questions, we point out that we have done H3 ChIP on the *HO* promoter at different time points during the cell cycle in synchronized cell population (Takahata et al. Mol Cell 34:405, 2009; Yu et al. Nucleic Acids Res 48:10877, 2020). In *cdc20* arrested cells, H3 has high ChIP signal near the Ash1 binding sites, and this signal goes down significantly at 20 minutes after release. This time coincides with the time of Ash1 binding. In addition, in a genome-wide screen for yeast pioneer factors (Yan et al. Mol Cell 71:294, 2018), we show that Ash1 cannot recognize nucleosome-embedded motifs and displace nucleosomes. Therefore, nucleosome depletion is likely required for its binding. Finally, our experiment in Figure 7 clearly shows that Ash1 binding on the *HO* promoter is strongly affected by nucleosome occupancy. Based on these findings, we would argue that Ash1 binding sites are occluded in most, if not all cells, and Swi5-induced nucleosome depletion is necessary to enable its binding.

2. An associated point is that the authors represent the nucleosomes as if they cover only 76 bp. This is often done to make the nucleosome map easier to interpret. However, it is a mis-representation of the data since the nucleosome covers 147 bp.

We have added a sentence to the Figure 1A legend indicating that the MNase-Seq were trimmed to show only the central 76-nt of each nucleosome so that their positions could be viewed more easily in the genome browser. MNase has a restricted cut pattern at A/T dinucleotides, generating nucleosomal fragments that can be larger or smaller than the actual nucleosome, based upon sequence differences. For this reason, edges of nucleosomes generated from MNase-Seq are inherently “fuzzy”, and viewing the central portion of the nucleosome is most informative.

3. Page 9: "An rpd3 null single mutant did not change expression of HO in the bulk population, but single-cell analysis demonstrated that HO was expressed in approximately 50% of the daughter cells." Could this be explained if the cells are producing the same amount of HO mRNA but, in rpd3-null cells, it is not being correctly partitioned between mother and daughter?

Since *HO* expression occurs after cell separation, we think it unlikely that the *HO* mRNA is not correctly partitioned between mother and daughter cells. We have added a sentence hypothesizing that mother/daughter differences differentially affect *HO* mRNA half-life and thus reduce the apparent contribution of daughter cell RNA to the bulk RNA total.

4. The ChIP-seq data for Ash1, Tup1 and Rpd3 appear to be of excellent quality (Figs. 4B and S4). The authors should show the ChIP-seq data for the HO gene.

We have added the ChIP-seq data for the *HO* gene to Figure S8.

5. The ChIP data as a function of the cell cycle show that the Swi5 activator binds first, then the Ash1 repressor (recruiting Tup1) and then HO transcription begins (Fig. 7A). Presumably, Ash1 is only binding and repressing in the daughter cells, while mother cells continue with activation. I think this could be stated more clearly in the Discussion.

The reviewer is correct that we could have been more clear in the Discussion. We have re-written a section emphasizing that while the large amount of Ash1 in daughters blocks activation, the smaller amount of Ash1 in mother cells does affect the regulation, making mother cell activation Gcn5-dependent.

6. The ChIP-seq data have been submitted to GEO but can't be accessed by the reviewers. I think that appropriate biological replicate experiments have been done, but I can't confirm that this is the case.

We apologize for the problem. I believe the GEO information was entered into the PLoS Genetics website for submitting a paper. Additionally, our cover letter to the editors listed the following:

The ChIP-Seq data at GEO is available to reviewers and journal editors with the following information:

To review GEO accession GSE158180:

Go to https://www.ncbi.nlm.nih.gov/geo/query/acc.cgi?acc=GSE158180

Enter token evkxawysbfgbpqb into the box

7. Figure 6: It is unclear what is plotted in the heat maps (define nucleosome density).

The Figure 6 legend has been re-written to clarify how the heat maps were generated.
